# Comparisons of the M1 genome segments and encoded μ2 proteins of different reovirus isolates

**DOI:** 10.1186/1743-422X-1-6

**Published:** 2004-09-23

**Authors:** Peng Yin, Natalie D Keirstead, Teresa J Broering, Michelle M Arnold, John SL Parker, Max L Nibert, Kevin M Coombs

**Affiliations:** 1 Department of Medical Microbiology and Infectious Diseases, University of Manitoba, Winnipeg, MB, R3E 0W3 Canada; 2 Thrasos Therapeutics, Hopkinton, MA 01748 USA; 3 Department of Pathobiology, Ontario Veterinary College, Guelph, ON, N1G 2W1 Canada; 4 Department of Microbiology and Molecular Genetics, Harvard Medical School, Boston, MA, 02115 USA; 5 Massachusetts Biologic Laboratories, Jamaica Plain, MA 02130-3597 USA; 6 Virology Training Program, Division of Medical Sciences, Harvard University, Cambridge, MA 02138 USA; 7 James A. Baker Institute for Animal Health, College of Veterinary Medicine, Cornell University, Ithaca, NY 14853 USA

## Abstract

**Background:**

The reovirus M1 genome segment encodes the μ2 protein, a structurally minor component of the viral core, which has been identified as a transcriptase cofactor, nucleoside and RNA triphosphatase, and microtubule-binding protein. The μ2 protein is the most poorly understood of the reovirus structural proteins. Genome segment sequences have been reported for 9 of the 10 genome segments for the 3 prototypic reoviruses type 1 Lang (T1L), type 2 Jones (T2J), and type 3 Dearing (T3D), but the M1 genome segment sequences for only T1L and T3D have been previously reported. For this study, we determined the M1 nucleotide and deduced μ2 amino acid sequences for T2J, nine other reovirus field isolates, and various T3D plaque-isolated clones from different laboratories.

**Results:**

Determination of the T2J M1 sequence completes the analysis of all ten genome segments of that prototype. The T2J M1 sequence contained a 1 base pair deletion in the 3' non-translated region, compared to the T1L and T3D M1 sequences. The T2J M1 gene showed ~80% nucleotide homology, and the encoded μ2 protein showed ~71% amino acid identity, with the T1L and T3D M1 and μ2 sequences, respectively, making the T2J M1 gene and μ2 proteins amongst the most divergent of all reovirus genes and proteins. Comparisons of these newly determined M1 and μ2 sequences with newly determined M1 and μ2 sequences from nine additional field isolates and a variety of laboratory T3D clones identified conserved features and/or regions that provide clues about μ2 structure and function.

**Conclusions:**

The findings suggest a model for the domain organization of μ2 and provide further evidence for a role of μ2 in viral RNA synthesis. The new sequences were also used to explore the basis for M1/μ2-determined differences in the morphology of viral factories in infected cells. The findings confirm the key role of Ser/Pro208 as a prevalent determinant of differences in factory morphology among reovirus isolates and trace the divergence of this residue and its associated phenotype among the different laboratory-specific clones of type 3 Dearing.

## Background

RNA viruses represent the most significant and diverse group of infectious agents for eukaryotic organisms on earth [1,2]. Virtually every RNA virus, except retroviruses, must use an RNA-dependent RNA polymerase (RdRp) to copy its RNA genome into progeny RNA, an essential step in viral replication and assembly. The virally encoded RdRp is not found in uninfected eukaryotic cells and therefore represents an attractive target for chemotherapeutic strategies to combat RNA viruses. A better understanding of the structure/function relationships of RNA-virus RdRps has been gained from recent determinations of X-ray crystal structures for several of these proteins, including the RdRps of poliovirus, hepatitis C virus, rabbit calicivirus, and mammalian orthoreovirus [3-6]. However, the diverse and complex functions and regulation of these enzymes, including their interactions with other viral proteins and cis-acting signals in the viral RNAs, determine that we have hardly scratched the surface for understanding most of them.

The nonfusogenic mammalian orthoreoviruses (reoviruses) are prototype members of the family *Reoviridae*, which includes segmented double-stranded RNA (dsRNA) viruses of both medical (rotavirus) and economic (orbivirus) importance (reviewed in [7-9]). Reoviruses have nonenveloped, double-shelled particles composed of eight different structural proteins encasing the ten dsRNA genome segments. Reovirus isolates (or "strains") can be grouped into three serotypes, represented by three commonly studied prototype isolates: type 1 Lang (T1L), type 2 Jones (T2J), and type 3 Dearing (T3D). Sequences have been reported for all ten genome segments of T1L and T3D, as well as for nine of the ten segments of T2J (all but the M1 segment) (*e.g.*, see [10,11]). Each of these segments encodes either one or two proteins on one of its strands, the plus strand. After cell entry, transcriptase complexes within the infecting reovirus particles synthesize and release full-length, capped plus-strand copies of each genome segment. These plus-strand RNAs are used as templates for translation by the host machinery as well as for minus-strand synthesis by the viral replicase complexes. The latter process produces the new dsRNA genome segments for packaging into progeny particles. The particle locations and functions of most of the reovirus proteins have been determined by a combination of genetic, biochemical, and biophysical techniques over the past 50 years (reviewed in [8]).

Previous studies have identified the reovirus λ3 protein, encoded by the L1 genome segment, as the viral RdRp [6,12-14]. Protein λ3 is a minor component of the inner capsid, present in only 10–12 copies per particle [15]. It has been proposed to bind to the interior side of the inner capsid, near the icosahedral fivefold axes, and recent work has precisely localized it there [16,17]. In solution, purified λ3 mediates a poly(C)-dependent poly(G)-polymerase activity, but it has not been shown to use virus-specific dsRNA or plus-strand RNA as template for plus- or minus-strand RNA synthesis, respectively [14]. This lack of activity with virus-specific templates suggests that viral or cellular cofactors may be required to make λ3 fully functional. Within the viral particle, where only viral proteins are known to reside, these cofactors are presumably viral in origin. The crystal structure of λ3 has provided substantial new information about the organization of its sequences and has suggested several new hypotheses about its functions in viral RNA synthesis and the possible roles of cofactors in these functions [6]. Notably, crystallized λ3 uses short viral and nonviral oligonucleotides as templates for RNA synthesis to yield short dsRNA products [6].

The reovirus μ2 protein has been proposed as a transcriptase cofactor, but it remains the most functionally and structurally enigmatic of the eight proteins found in virions. Like λ3, μ2 is a minor component of the inner capsid, present in only 20–24 copies per particle [15]. It is thought to associate with λ3 in the particle interior, in close juxtaposition to the icosahedral fivefold axes, but has not been precisely localized there [16,17]. A recent study has shown that purified μ2 and λ3 can interact in vitro [18]. The M1 genome segment that encodes μ2 is genetically associated with viral strain differences in the in vitro transcriptase and nucleoside triphosphatase (NTPase) activities of viral particles [19,20]. Recent work with purified μ2 has shown that it can indeed function in vitro as both an NTPase and an RNA 5'-triphosphatase [18]. The μ2 protein has also been shown to bind RNA and to be involved in formation of viral inclusions, also called "factories", through microtubule binding in infected cells [18,21-23]. Nevertheless, its precise function(s) in the reovirus replication cycle remain unclear. Other studies have indicated that the μ2-encoding M1 segment genetically determines the severity of cytopathic effect in mouse L929 cells, the frequency of myocarditis in infected mice, the levels of viral growth in cardiac myocytes and endothelial cells, the degree of organ-specific virulence in severe combined immunodeficiency mice, and the level of interferon induction in cardiac myocytes [24-29]. The complete sequence of the M1 segment has been reported for both T1L and T3D [23,30,31]. However, computer-based comparisons of the M1 and μ2 sequences to others in GenBank have previously failed to show significant homology to other proteins, so that no clear indications of μ2 function have come from that approach. Nevertheless, small regions of sequence similarity to NTP-binding motifs have been identified near the middle of μ2, and recent work has indicated that mutations in one of these regions indeed abrogates the triphosphatase activities of μ2 [18,20].

For this study, we performed nucleotide-sequence determinations of the M1 genome segments of reovirus T2J, nine other reovirus field isolates, and reovirus T3D clones obtained from several different laboratories. The determination of the T2J M1 sequence completes the sequence determination of all ten genome segments of that prototype strain. We reasoned that comparisons of additional M1 and μ2 sequences may reveal conserved features and/or regions that provide clues about μ2 structure and function. The findings provide further evidence for a role of μ2 in viral RNA synthesis. We also took advantage of the newly available sequences to explore the basis for M1/μ2-determined strain differences in the morphology of viral factories in reovirus-infected cells.

## Results and Discussion

### M1 nucleotide and μ2 amino acid sequences of reovirus T2J and nine other field isolates

**Figure 1:**
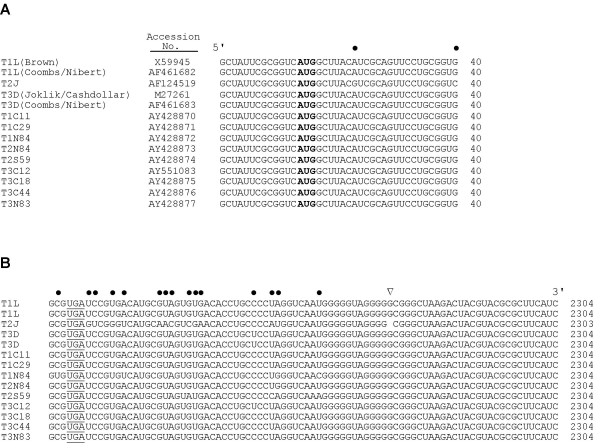
Sequences near the 5' (A) and 3' (B) ends of the M1 plus strands of 14 reovirus isolates. The start and stop codons are indicated by bold and underline, respectively. The one-base deletion in the 3' noncoding region of the T2J sequence is indicated by a triangle. Positions at which at least one sequence differs from the others are indicated by dots. GenBank accession numbers for corresponding sequences are indicated between the clones' names and 5' sequences in "A". Clones are: T1L (type 1, Lang), T1C11 (type 1, clone 11), T1C29 (type 1, clone 29), T1N84 (type 1, Netherlands 1984), T2J (type 2, Jones), T2N84 (type 2, Netherlands 1984), T2S59 (type 2, simian virus 59), T3D (type 3, Dearing), T3C12 (type 3, clone 12), T3C18 (type 3, clone 18), T3C44 (type 3, clone 44), and T3N83 (type 3, Netherlands 1983). T1L clones were obtained from Dr. E.G. Brown (Brown) or our laboratories (Coombs/Nibert). T3D clones were obtained from Drs. W.K. Joklik, L.W. Cashdollar (Joklik/Cashdollar) and our laboratories (Coombs/Nibert).

**Figure 2:**
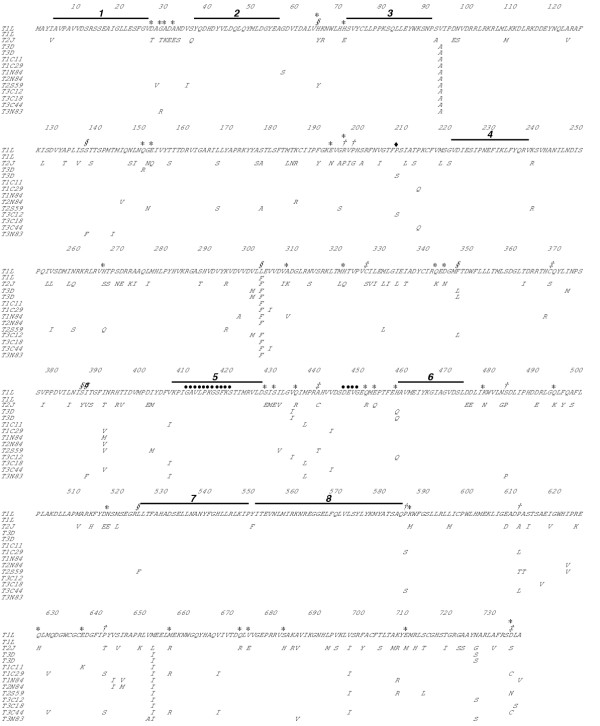
Alignment of the deduced μ2 amino acid sequences of T1L, T2J, T3D, and various field isolates. The single-letter amino acid code is used, and only the T1L μ2 sequence from the Brown laboratory is shown in its entirety. For other isolates, only those amino acids that differ from this T1L sequence are shown. Clones arranged in same order as in Fig. 1; the second T1L μ2 sequence is from the Nibert and Coombs laboratories, the first T3D μ2 sequence is from the Joklik and Cashdollar laboratories, and the second T3D μ2 sequence is from the Nibert and Coombs laboratories. Amino acid positions are numbered above the sequences. Some symbols represent various nonconservative changes among the isolates: *, change involving a charged residue; § change involving an aromatic residue; †, change involving a proline residue; ‡, change involving a cysteine residue. Residue 208, which has been previously shown to affect microtubule association by μ2, is indicated by a filled diamond. Residues 410–420 and 446–449, which have been previously identified as NTP-binding motifs are indicated by filled circles. Consecutive runs of wholly conserved residues ≥ 15 amino acids in length are indicated by the lines numbered 1 to 8.

We determined the nucleotide sequence of the M1 genome segment of reovirus T2J to complete the sequencing of that isolate's genome. T2J M1 was found to be 2303 base pairs in length (GenBank accession no. AF124519) (Table 1). This is one shorter than the M1 segments of reoviruses T1L and T3D [23,30,31], due to a single base-pair deletion in T2J corresponding to position 2272 in the 3' nontranslated region of the T1L and T3D plus strands (Fig. 1, Table 1). Like those of T1L and T3D, the T2J-M1 plus strand contains a single long open reading frame, encoding a μ2 protein of 736 amino acids (Fig. 2, Table 1), having the same start and stop codons (Fig. 1), and having a 5' nontranslated region that is only 13 nucleotides in length (Table 1). Because of the single-base deletion described above, the 3' nontranslated region of the T2J M1 plus strand is only 82 nucleotides in length, compared to 83 for T1L and T3D (Table 1). Regardless, M1 has the longest 3' nontranslated region of any of the genome segments of these viruses, the next longest being 73 nucleotides in S3 (reviewed in [32]).

**Table 1:** Features of M1 genome segments and μ2 proteins from different reovirus isolates

	Reovirus isolate^a^												
	
M2 or μ2 property^b^	T1L^c^	T2J	T3D^d^	T3D^e^	T1C11	T1C29	T1N84	T2N84	T2S59	T3C12	T3C18	T3C44	T3N83
Accession no.:	X59945	AF124519	M27261	AF461683	AY428870	AY428871	AY428872	AY428873	AY428874	AY551083	AY428875	AY428876	AY428877
	
total nuc	2304	2303	2304	2304	2304	2304	2304	2304	2304	2304	2304	2304	2304
5' NTR	13	13	13	13	13	13	13	13	13	13	13	13	13
3' NTR	83	82	83	83	83	83	83	83	83	83	83	83	83
total AA	736	736	736	736	736	736	736	736	736	736	736	736	736
mass (kDa)	83.3	84.0	83.3	83.2	83.2	83.3	83.4	83.3	83.5	83.2	83.3	83.3	83.4
pI	6.92	7.44	6.98	6.89	7.10	7.09	6.98	6.92	6.96	6.89	6.92	7.09	7.01
Asp+Glu	85	84	85	85	84	84	85	85	84	85	85	84	85
Arg+Lys+His	102	105	102	101	103	103	102	102	100	101	102	103	103

^a ^Abbreviations defined in text.

^b ^nuc, nucleotides; NTR, nontranslated region; AA, amino acids; pI, isoelectric point.

^c ^All indicated values are the same for the T1L M1 and μ2 sequences obtained for the Brown laboratory clone [31] (indicated GenBank accession number), the Nibert laboratory clone [23]; GenBank accession no. AF461682), and the Coombs laboratory clone (this study).

^d ^T3D M1 and μ2 sequences for the Joklik laboratory clone [30] (indicated GenBank accession number), and the Cashdollar laboratory clone [23]; GenBank accession no. AF461684).

^e ^T3D M1 and μ2 sequences for the Nibert laboratory clone [23] and the Coombs laboratory clone (this study).

To gain further insights into μ2 structure/function relationships, we determined the M1 nucleotide sequences of nine other reovirus field isolates [33,34]. The M1 segments of each of these viruses were found to be 2304 base pairs in length (GenBank accession nos. AY428870 to AY428877 and AY551083), the same as T1L and T3D M1 (Fig. 1). Like those of T1L, T2J, and T3D, the M1 plus strand from each of the field isolates contains a single long open reading frame, again encoding a μ2 protein of 736 amino acids (Fig. 2) and having the same start and stop codons (Fig. 1). Their 5' and 3' nontranslated regions are therefore the same lengths as those of T1L and T3D M1 (Table 1). As part of this study, we also determined the M1 nucleotide sequences of the reovirus T1L and T3D clones routinely used in the Coombs laboratory. We found these sequences to be identical to those recently reported for the respective Nibert laboratory clones [23].

### Further comparisons of the M1 nucleotide sequences

The T2J M1 genome segment shares 71–72% homology with those of both T1L and T3D (Table 2). This makes T2J M1 the most divergent of all nonfusogenic mammalian orthoreovirus genome segments examined to date, with the exception of the S1 segment, which encodes the attachment protein σ1 and which shows less than 60% nucleotide sequence homology between serotypes [35,36]; reviewed in [11]. In contrast, the homology between T1L and T3D M1 is ~98%, among the highest values seen to date between reovirus genome segments from distinct field isolates [11,31,34,37-39].

**Table 2:** Pairwise comparisons of M1 genome segment and μ2 protein sequences from different reovirus isolates

	Identity (%) compared with reovirus isolate^a^													
	
Virus isolate	T1L^b^	T1L^c^	T2J	T3D^d^	T3D^e^	T1C11	T1C29	T1N84	T2N84	T2S59	T3C12	T3C18	T3C44	T3N83
T1L^b^	--	99.9^f^	80.8	98.6	98.8	99.2	98.0	98.4	98.8	96.3	98.8	99.0	98.0	98.2
T1L^c^	**99.9**^f^	--	81.0	98.8	98.9	99.3	98.1	98.5	98.9	96.2	98.9	99.2	98.1	98.4
T2J	**71.6**	**71.6**	--	80.0	80.2	80.4	80.3	80.2	80.4	81.5	80.2	80.3	80.3	80.4
T3D^d^	**97.8**	**97.9**	**70.9**	--	99.6	98.6	97.4	97.8	98.2	95.5	99.6	98.5	97.4	98.0
T3D^e^	**97.9**	**98.0**	**71.0**	**99.7**	--	98.8	97.6	98.0	98.4	95.7	100	98.6	97.6	98.1
T1C11	**98.7**	**98.7**	**71.3**	**97.1**	**97.1**	--	98.0	98.4	98.8	96.1	98.8	99.6	98.0	98.8
T1C29	**96.3**	**96.4**	**71.1**	**95.8**	**95.8**	**95.5**	--	97.3	97.8	95.7	97.6	97.8	100	97.0
T1N84	**96.3**	**96.3**	**70.8**	**95.7**	**95.8**	**95.9**	**94.5**	--	98.5	95.7	98.0	98.2	97.3	97.4
T2N84	**97.1**	**97.1**	**71.0**	**96.5**	**96.6**	**96.7**	**95.4**	**96.5**	--	96.2	98.4	98.6	97.8	97.8
T2S59	**89.8**	**89.9**	**71.3**	**89.2**	**89.3**	**89.2**	**89.4**	**89.1**	**89.7**	--	95.7	95.9	95.7	95.1
T3C12	**97.8**	**97.9**	**71.0**	**99.7**	**99.9+**	**97.2**	**95.7**	**95.7**	**96.6**	**89.3**	--	98.6	97.6	98.1
T3C18	**98.8**	**98.9**	**71.2**	**97.3**	**97.4**	**99.4**	**95.8**	**95.8**	**96.8**	**89.4**	**97.4**	--	97.8	98.6
T3C44	**96.5**	**96.6**	**71.1**	**95.9**	**95.9**	**95.7**	**99.7**	**94.6**	**95.5**	**89.4**	**95.9**	**96.0**	--	97.0
T3N83	**97.7**	**97.8**	**71.4**	**96.4**	**96.4**	**98.6**	**94.7**	**94.9**	**95.8**	**88.5**	**96.4**	**98.4**	**95.0**	--

^a ^Abbreviations defined in text.

^b ^T1L M1 and μ2 sequences for the Brown laboratory clone [31]; GenBank accession no. X59945).

^c ^T1L M1 and μ2 sequences for the Nibert laboratory clone [23]; GenBank accession no. AF461682) and the Coombs laboratory clone (this study).

^d ^T3D M1 and μ2 sequences for the Joklik laboratory clone [30]; GenBank accession no. M27261), and the Cashdollar laboratory clone [23]; GenBank accession no. AF461684).

^e ^T3D M1 and μ2 sequences for the Nibert laboratory clone [23]; GenBank accession no. AF461683) and the Coombs laboratory clone (this study).

^f ^Values for M1-gene sequence comparisons are shown below the diagonal, in bold; values for μ2-protein sequence comparisons are shown above the diagonal.

**Figure 3:**
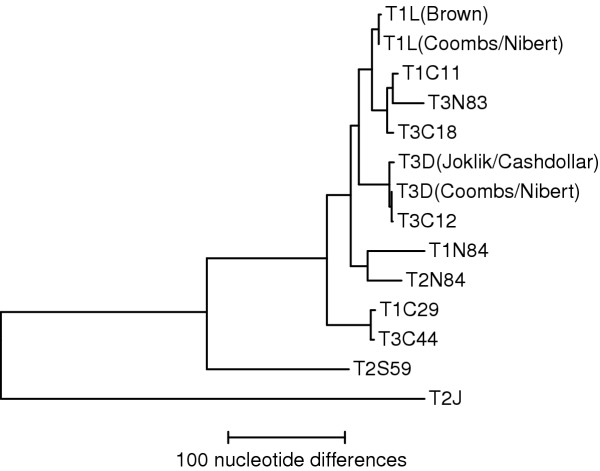
Most parsimonious phylogenetic tree based on the M1 nucleotide sequences of the different reoviruses. Sequences for T1L and T3D clones from different laboratories are shown (laboratory source(s) in parentheses). Horizontal lines are proportional in length to nucleotide substitutions.

The M1 genome segments of the nine other reovirus isolates examined in this study are much more closely related to those of T1L and T3D than to that of T2J (Table 2), as also clearly indicated by phylogenetic analyses (Fig. 3 and data not shown). Such greater divergence of the gene sequences of T2J has been observed to date with other segments examined from multiple reovirus field isolates [11,34,37-39]. Type 2 simian virus 59 (T2S59) has the next most broadly divergent M1 sequence, but it is no more similar to the M1 sequence of T2J than it is to that of the other isolates (Table 2, Fig. 3). In sum, the results of this study provided little or no evidence for divergence of the M1 sequences along the lines of reovirus serotype (Fig. 3), consistent with independent reassortment and evolution of the M1 and S1 segments in nature. Upon considering the sources of these isolates [34], the results similarly provided little or no evidence for divergence of the M1 sequences along the lines of host, geographic locale, or date of isolation (Fig. 3). These findings are consistent with ongoing exchange of M1 segments among reovirus strains cocirculating in different hosts and locales. Similar conclusions have been indicated by previous studies of other genome segments from multiple reovirus field isolates [11,34,37-39]. The M1 nucleotide sequence of type 3 clone 12 (T3C12) is almost identical to that of the T3D clone in use in the Coombs and Nibert laboratories, with only a single silent change (U→C) at plus-strand position 1532 (*i.e., *99.9+% homology). However, several of the T3C12 genome segments show distinguishable mobilities in polyacrylamide gels (data not shown), confirming that T3C12 is indeed a distinct isolate.

### Further comparisons of the μ2 protein sequences

The T2J μ2 protein shares 80–81% homology with those of both T1L and T3D (Table 2, Fig. 2). Consistent with the M1 nucleotide sequence results, this makes T2J μ2 the most divergent of all nonfusogenic mammalian orthoreovirus proteins examined to date, with the exception of the S1-encoded σ1 and σ1s proteins, which show less than 55% amino acid sequence homology between serotypes [35,36]; reviewed in [11]. In contrast, the homology between T1L and T3D μ2 approaches 99%, among the highest values seen to date between reovirus genome segments from distinct isolates [11,31,34,37-39]. Also consistent with the M1 nucleotide sequence results, the μ2 proteins of the nine other reovirus isolates examined in this study are much more closely related to those of T1L and T3D than to that of T2J (Table 2, Fig. 3), affirming the divergent status of the T2J μ2 protein. The μ2 protein sequence of T3C12 is identical to that of the T3D clone in use in the Coombs and Nibert laboratories. In addition, the μ2 protein sequence of T1C29 is identical to that of T3C44. These are the first times that reovirus proteins from distinct isolates have been found to share identical amino acid sequences [11,32,34,37-39], reflecting the high degree of μ2 conservation.

The encoded μ2 proteins of the twelve reovirus isolates are all calculated to have molecular masses between 83.2 and 84.0 kDa, and isoelectric points between 6.89 and 7.44 pH units (Table 1). This range of isoelectric points is the largest yet seen among reovirus proteins other than σ1s [11], but is largely attributable to the divergent value of T2J μ2 (others range only from 6.89 to 7.10). The substantially higher isoelectric point of T2J μ2 is explained by it containing a larger number of basic residues (excess arginine) than do the other isolates (Table 1).

**Figure 4:**
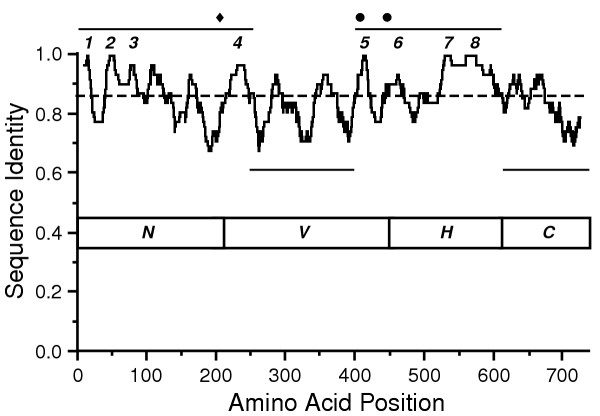
Window-averaged scores for sequence identity among the T1L, T2J, and T3D μ2 proteins. Identity scores averaged over running windows of 21 amino acids and centered at consecutive amino acid positions are shown. The global identity score for the three sequences is indicated by the dashed line. Two extended areas of greater-than-average sequence variation are marked with lines below the plot. Two extended areas of greater-than-average sequence conservation are marked with lines above the plot. Eight regions of ≥ 15 consecutive residues of identity among all twelve μ2 sequences from Fig. 2, as discussed in the text, are numbered above the plot. The Ser/Pro208 determinant of microtubule binding is marked with a filled diamond. The two putative NTP-binding motifs are marked with filled circles.

Comparisons of the twelve μ2 sequences showed eight highly conserved regions, each containing ≥ 15 consecutive residues that are identical in all of the isolates (Fig. 2). The highly conserved regions are clustered in two larger areas of μ2, spanning approximately amino acids 1–250 and amino acids 400–610. Conserved region 5 in the 400–610 area encompasses the more amino-terminal of the two NTP-binding motifs in μ2 (Fig. 2) [18,20]. The other NTP-binding motif is also wholly conserved, but within a smaller consecutive run of conserved residues. The region between the two motifs is notably variable (Fig. 2). Conserved region 5 also contains the less conservative of the two amino acid substitutions in T1L-derived temperature-sensitive (*ts*) mutant *tsH11.2 *(Pro414→His) [40]. The pattern of conserved and variable areas of μ2 was also seen by plotting scores for sequence identity in running windows over the protein length (*e.g.*, [32]). In addition to the conserved regions described above, areas of greater than average variation are evident in this plot, spanning approximately amino acids 250–400 and 610–736 (the carboxyl terminus) (Fig. 4). The 250–400 area is notable for regularly oscillating between conserved and variable regions (Fig. 4). The two large areas of greater-than-average sequence conservation, spanning approximately amino acids 1–250 and 400–610 (Fig. 4), are likely to be involved in the protein's primary function(s). The more variable, 250–400 area between the two conserved ones might represent a hinge or linker of mostly structural importance.

**Figure 5:**
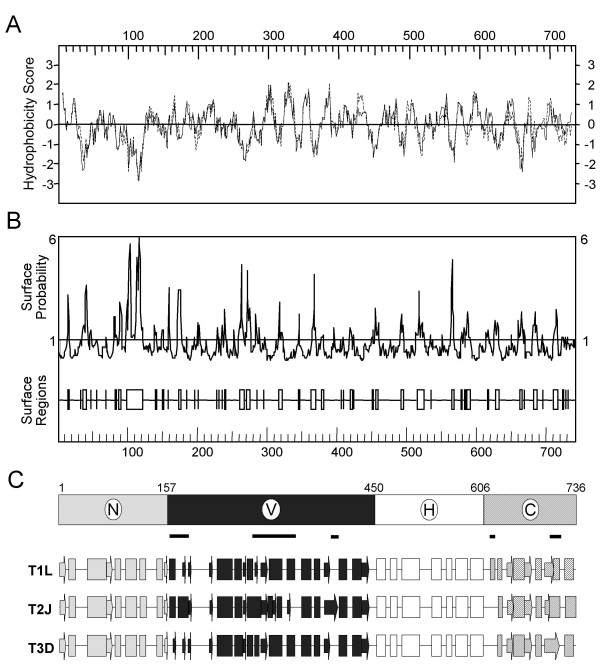
Secondary structure predictions of μ2 protein. (A) Hydropathicity index predictions of T2J (- - -) and T1L (-----) μ2 proteins, superimposed to accentuate similarities and differences. Hydropathy values were determined by the Kyte-Doolittle method [72], using DNA Strider 1.2, a window length of 11, and a stringency of 7. (B) Surface probability predictions of the T2J μ2 protein, determined as per Emini et al. [73], using DNASTAR. The predicted surface probability profiles of T1L and T3D (not shown) were identical to T2J. (C) Locations of α-helices and β-sheets were determined by the PHD PredictProtein algorithms [74], and results were graphically rendered with Microsoft PowerPoint software., α-helix;., β-sheet;—, turn. Differences in fill pattern correspond to arbitrary division of protein into four regions; N, amino terminal; V, variable; H, helix-rich; C, carboxyl terminal. The locations of variable regions are indicated by the thick lines under the domain representation.

As indicated earlier, μ2 is one of the most poorly understood reovirus proteins, from both a functional and a structural point of view. For example, atomic structures are available for seven of the eight reovirus structural proteins, with μ2 being the missing one. Thus, in an effort to refine the model for μ2 structure/function relationships based on regional differences, we obtained predictions for secondary structures, hydropathy, and surface probability. PHD PredictProtein algorithms suggest that μ2 can be divided into four approximate regions characterized by different patterns of predicted secondary structures (Fig. 5C). An amino-terminal region spans to residue 157, a "variable" region spans residues 157 to 450, a "helix-rich" region spans residues 450 to 606, and a carboxyl-terminal region spans the sequences after residue 606. The amino-terminal region contains six predicted α-helices and three predicted β-strands, and is highly conserved across all twelve μ2 sequences. The "variable" region is the most structurally complex and contains numerous interspersed α-helices and β-strands. The "helix-rich" region contains seven α-helices and is highly conserved across all twelve μ2 sequences. The carboxyl-terminal region varies across all three serotypes. Overall, the μ2 protein is predicted to be 48% α-helical and 14% β-sheet in composition, making it an "α-β " protein according to the CATH designation [41]. Interestingly, most tyrosine protein kinases with SH_2 _domains are also "α-β " proteins by this designation. The T1L and T3D μ2 hydropathy profiles were identical to each other. Both show numerous regions of similarity to the hydropathy profile of the T2J μ2. However, there also are several distinct differences between the T1L and T2J profiles (Fig. 5). Alterations in amino acid charge at residues 32, 430 to 432, and 673 in the T2J sequence account for the major differences in hydrophobicity between T2J and the other serotypes. In addition, the carboxyl-terminal 66 residues show multiple differences in hydropathy. The surface probability profiles of each of the three serotype's μ2 proteins are identical (Fig. 5) and show numerous regions that are highly predicted to be exposed at the surface of the protein as well as regions predicted to be buried.

The MOTIF and FingerPRINTScan programs were used to compare the highly conserved regions of μ2 with other sequences in protein data banks (ProSite, Blocks, and ProDomain). The results revealed that several of the conserved regions in μ2 share limited similarities with members of the DNA polymerase A family and with the SH_2 _domain of tyrosine kinases. The sequence YEAgDV in μ2, located in conserved region 2 (Fig. 2), is similar to the "YAD" motif of DNA polymerase A from a number of different bacteria (*e.g.*, YEADDV in *Deinococcus radiodurans*). The YAD motif is located in the exonuclease region of DNA polymerase A, a region which also functions as an NTPase and enhances the rate of DNA polymerization [42]. The SH_2 _domain of tyrosine kinases was the highest score hit for the conserved regions of μ2 with FingerPRINTScan. Four of the five motifs in the 100 amino acid SH_2 _domain matched the μ2 sequence. The SH_2 _domain mediates protein-protein interactions through its capacity to bind phosphotyrosine [43]. The protein motifs found by focusing on the conserved regions of μ2 provide supportive evidence that this protein is involved in nucleotide binding and metabolism. However, the described similarities did not match with greater than 90% certainty and no other significant homologies were detected. The inability to identify higher-scoring GenBank similarities, first noted when sequences of the T3D and T1L M1 genes were reported [30,31] attests to the uniqueness of this minor core protein.

### Biochemical confirmations

**Figure 6:**
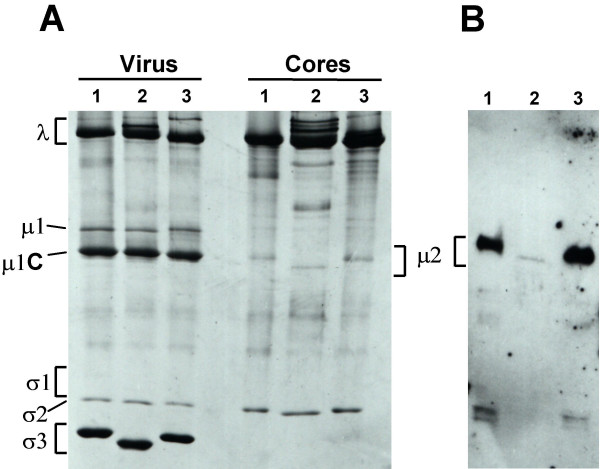
SDS-PAGE and immunoblot analyses of virion and core particles. Proteins from gradient-purified T1L (1), T2J (2), and T3D (3) particles were resolved in 5–15% SDS-polyacrylamide gels as detailed in Materials and methods. Gels were then fixed and stained with Coomassie Brilliant Blue R-250 and silver (A). Alternatively, proteins from the gels were transferred to nitrocellulose, probed with anti-μ2 antiserum (polyclonal antibodies raised against T1L μ2, kindly provided by E. G. Brown), and detected by chemiluminescence (B). Virion proteins are indicated to the left of panel A, except for μ2, which is indicated between the panels.

In an effort to provide biochemical confirmation of the predicted variation in the different isolates' μ2 proteins, we analyzed the T1L, T2J, and T3D proteins by sodium dodecyl sulfate-polyacrylamide gel electrophoresis (SDS-PAGE) and immunoblotting. Despite the slightly larger molecular mass calculated from its sequence (Table 1), T2J μ2 displayed a slightly smaller relative molecular weight on gels than T1L and T3D μ2 (Fig. 6A). This aberrant mobility may reflect the higher isoelectric point of T2J μ2 (Table 1). Polyclonal anti-μ2 antibodies that had been raised against purified T1L μ2 [44] reacted strongly with both T1L and T3D μ2, but only weakly with T2J μ2 (Fig. 6B), despite equal band loading as demonstrated by Ponceau S staining. These antibody cross-reactivities correlated well with the predicted protein homologies (Table 2).

### Factory morphologies among reovirus field isolates

**Figure 7:**
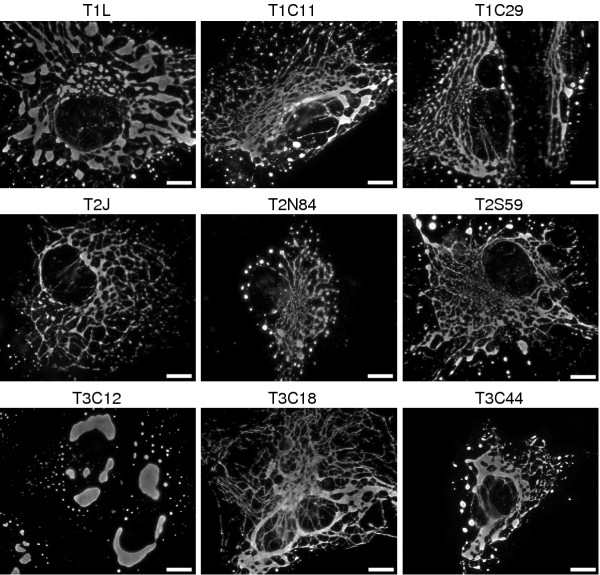
Viral factory morphology as demonstrated by the distribution of μNS in cells infected with various reovirus isolates. CV-1 cells were infected at 5 PFU/cell with the isolate indicated above each panel, fixed at 18 h p.i., and immunostained with μNS-specific rabbit IgG conjugated to Alexa 594. Size bars, 10 μm.

We took advantage of the new M1/μ2 sequences to extend analysis of the role of μ2 in determining differences in viral factory morphology among reovirus isolates [23]. Sequence variation at μ2 residue Pro/Ser208 was previously indicated to determine the different morphologies of T1L and T3D factories: Pro208 is associated with microtubule-anchored filamentous factories, as in T1L and the Cashdollar laboratory clone of T3D, whereas Ser208 is associated with globular factories, as in the Nibert laboratory clone of T3D [23]. For the previous study we had already examined the factories of T2J and some of the nine other isolates used for M1 sequencing above. We nonetheless newly examined the factories of all ten isolates in the present study, using the same stocks used for sequencing. T3C12 was the only one of these isolates that formed globular factories; the remainder, including T2J, formed filamentous factories (Fig. 7, Table 4). This finding is consistent with the fact that T3C12 is the only one of these isolates that has a serine at μ2 residue 208, like T3D from the Nibert laboratory; the remainder, like T1L and T3D from the Cashdollar laboratory, have a proline there (Fig. 2, Table 4) [23]. Thus, although the results identify no additional μ2 residues that may influence factory morphology, they are consistent with the identification of Pro/Ser208 as a prevalent determinant of differences in this phenotype among reovirus isolates.

**Table 4:** Properties of different reovirus isolates

Virus isolate^a^	Virus factory morphology^b^	Amino acid at μ2 position 208
T1L	filamentous^c^	Pro^c^
T2J	filamentous^d^	Pro
T3D^e^	filamentous^c^	Pro^c^
T3D^f^	globular^c^	Ser^c^
T1C11	filamentous	Pro
T1C29	filamentous	Pro
T1N84	filamentous^d^	Pro
T2N84	filamentous^d^	Pro
T2S59	filamentous^d^	Pro
T3C12	globular^d^	Ser
T3C18	filamentous^d^	Pro
T3C44	filamentous	Pro
T3N83	filamentous^d^	Pro

^a ^Abbreviations defined in the text.

^b ^Determined by immunofluorescence microscopy as described in the text.

^c ^Reported in Parker et al. [23].

^d ^Reported in supplementary data of Parker et al. [23].

^e ^T3D clone from the Cashdollar laboratory.

^f ^T3D clone from the Nibert laboratory.

### Factory morphologies and M1/μ2 sequences of other T3D and T3D-derived clones

**Figure 8:**
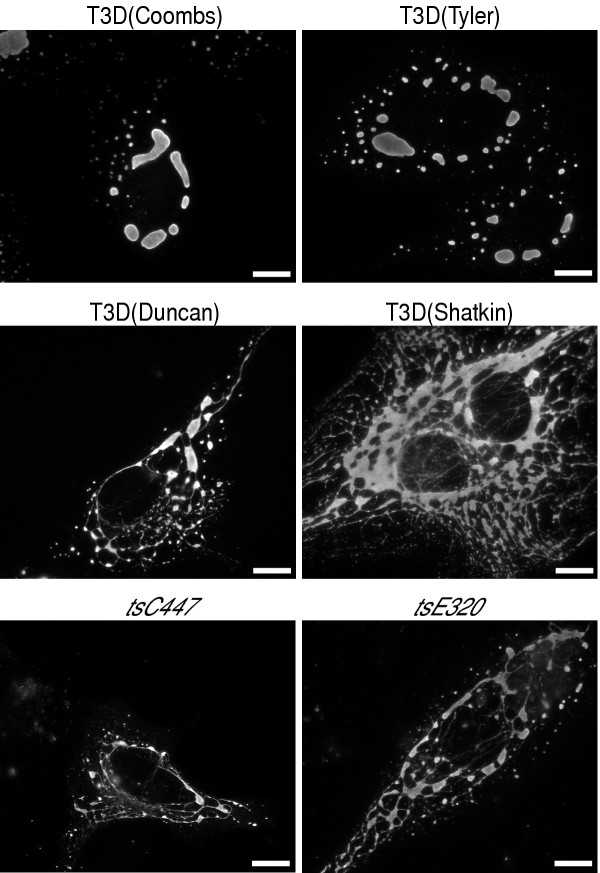
Viral factory morphology as demonstrated by the distribution of μNS in cells infected with T3D clones obtained from different laboratories or with T3D-derived *ts *clones. Laboratory sources are indicated in parentheses. CV-1 cells were infected at 5 PFU/cell with the clone indicated above each panel, fixed at 18 h p.i., and immunostained with μNS-specific rabbit IgG conjugated to Alexa 488. Size bars, 10 μm.

T3D clones from the Nibert and Cashdollar laboratories have been shown to exhibit different factory morphologies based on differences in the microtubule-binding capacities of their μ2 proteins and the presence of either serine or proline at μ2 residue 208 [23]. We took the opportunity in this study to examine additional T3D clones. The clones from some laboratories formed globular factories in infected cells whereas those from other laboratories or the American Type Culture Collection formed filamentous factories (Fig. 8, Table 5). T3D-derived *ts *mutants *tsC447*, *tsE320*, and *tsG453 *[45] formed filamentous factories (Fig. 8, Table 5). Other *ts *mutants were not examined; however, [46] have shown evidence that *tsF556 *[45] forms filamentous factories as well.

**Table 5:** Properties of different T3D and T3D-derived clones

			Positions of variation in T3D μ2			
			
Virus isolate	Laboratory source	Virus factory morphology				
			150	208	224	372
T3D	Nibert^a^	globular^b^	Gln	Ser^b^	Glu	Ile
T3D	Coombs^a^	globular	Gln	Ser	Glu	Ile
T3D	Schiff^a^	globular	Gln	Ser	Glu	Ile
T3D	Tyler^a^	globular	Gln	Ser	Glu	Ile
T3D	Cashdollar^c^	filamentous^b^	Arg	Pro^b^	Glu	Met
T3D	Duncan^c^	filamentous	Arg	Pro	Glu	Met
T3D	Shatkin	filamentous	Gln	Pro	Ala	Ile
T3D	ATCC	filamentous	Gln	Pro	Glu	Ile
*tsC447*	Coombs^c^	filamentous	Gln	Pro	Glu	Ile
*tsE320*	Coombs^c^	filamentous	Gln	Pro	Glu	Ile
*tsG453*	Coombs^c^	filamentous	Gln	Pro	Glu	Ile

^a ^Origin traceable to B. N. Fields laboratory.

^b ^Reported in Parker et al. [23].

^c ^Origin traceable to W. K. Joklik laboratory; derived from T3D; sequences of *tsC447 *(GenBank accession no. AY428878), *tsE320*, and *tsG453 *are identical.

We additionally determined the M1 sequences of the wild-type and *ts *T3D clones newly tested for factory morphology. All clones with globular factories have a serine at μ2 position 208 whereas all those with filamentous factories have a proline there (Table 5). These findings provide further evidence for the influence of residue 208 on this phenotypic difference.

All wild-type T3D clones with globular factories were recently derived from a Fields laboratory parent whereas all wild-type or *ts *T3D clones with filamentous factories were derived from parents in other laboratories. (Although extensively characterized by both Fields (*e.g.*, [47,48]) and Joklik (*e.g.*, [49,50]), the original T3D-derived *ts *mutants in groups A through G were generated in the Joklik laboratory [45]). This correlation suggests that formation of filamentous factories is the ancestral phenotype of reovirus T3D and that the Ser208 mutation in T3D μ2 was established later, in the Fields laboratory. As we noted in a previous study [23], several other laboratories reported evidence for filamentous T3D factories in the 1960's (*e.g.*, [51,52]), following its isolation in 1955 [53]. Since microtubules were noted to be commonly associated with T3D factories in Fields laboratory publications from as late as 1973 [54], but not in one from 1979 [55], the μ2 Ser208 mutation was probably established in, or introduced into, that laboratory during the middle 1970's. Investigators should be alert to these different lineages of T3D and their derivatives for genetic studies. For example, reassortant 3HA1 [56] contains a T3D M1 genome segment derived from clone *tsC447*, and its factory phenotype is filamentous (data not shown).

### Additional genome-wide comparisons of T1L, T2J, and T3D

Several types of genome-wide comparisons of T1L, T2J, and T3D have been reported previously [11]. For this study we examined the positions and types of nucleotide mismatches in these prototype isolates in order to gain a more comprehensive view of the evolutionary divergence of their protein-coding sequences. Most mismatches between T2J and either T1L or T3D segments, ~68%, are in the third codon base position, while ~21% are in the first position and ~11% are in the second position. Each of these mismatch percentages was converted to an evolutionary divergence value by multiplying mismatch percentage by 1.33 [31] (Table 3). These values have been used to argue that the homologous T1L and T3D genome segments diverged from common ancestors at different times in the past, with the M1 and L3 segments having diverged most recently and the M2, S1, S2, and S3 segments having diverged longer ago [31]. The consistently high values for divergence at third codon base positions among pairings with T2J genome segments (Table 3) indicate that all ten T2J segments diverged from common ancestors substantially before their respective T1L and T3D homologs. Relative numbers of synonymous and nonsynonymous nucleotide changes identified in pairwise comparisons of the coding sequences of these isolates (Table 3) support the same conclusion.

**Table 3:** Pairwise comparisons of variation at different codon positions in reovirus genome segments

		Variation (%) in the long open reading frame of genome segment								
		
Codon position	Isolate pair	L1	L2	L3	M1	M2	M3	S2	S3	S4
first^a^	T1L:T2J	16.9	19.9	12.2	24.6	11.1	25.3	13.7	25.5	13.1
	T2J:T3D	16.7	20.4	12.7	26.1	10.7	25.0	14.0	25.5	13.9
	T1L:T3D	2.4	15.4	1.4	1.5	6.0	7.6	6.1	6.6	4.0
second^a^	T1L:T2J	5.3	8.0	3.3	11.8	1.7	10.0	4.1	8.4	5.1
	T2J:T3D	5.1	7.5	3.2	11.8	1.7	9.6	4.1	8.0	5.5
	T1L:T3D	0.8	3.5	0.3	0.4	2.1	2.0	0.0	2.2	1.1
third^a^	T1L:T2J	77.1	83.7	79.4	80.1	81.5	81.2	74.0	79.1	73.8
	T2J:T3D	76.7	77.4	79.1	81.0	82.7	83.0	73.0	73.9	76.7
	T1L:T3D	12.9	76.1	7.5	6.5	53.3	39.2	53.6	48.1	21.9
syn.^b^	T1L:T2J	88.3	90.2	89.6	85.8	90.0	87.1	83.8	90.2	81.9
	T2J:T3D	87.5	84.2	89.3	87.0	89.3	89.8	83.6	85.4	84.2
	T1L:T3D	15.0	85.9	8.8	7.9	59.3	46.4	63.1	58.2	25.8
nonsyn.^b^	T1L:T2J	5.9	9.1	3.8	12.6	2.6	11.8	4.8	10.2	6.2
	T2J:T3D	5.9	8.9	3.9	13.1	3.2	11.5	4.7	9.6	6.8
	T1L:T3D	0.8	5.0	0.3	0.5	1.2	2.0	0.7	1.3	1.3
cons.^c^	T1L:T2J	60.0	66.3	57.1	63.8	50.0	60.6	50.0	60.8	73.5
		**5.0**	**8.7**	**2.5**	**12.2**	**1.3**	**10.7**	**2.9**	**8.5**	**6.8**
	T2J:T3D	62.7	64.5	56.1	64.6	65.2	60.5	52.0	60.8	71.1
		**5.1**	**8.6**	**2.5**	**12.9**	**2.1**	**10.0**	**3.1**	**8.5**	**7.4**
	T1L:T3D	36.4	77.4	88.9	80.0	50.0	62.5	100	40.0	63.6
		**0.6**	**5.6**	**0.6**	**1.1**	**1.1**	**2.8**	**1.2**	**1.0**	**1.9**
noncon.^c^	T1L:T2J	18.1	10.7	17.9	17.0	11.1	18.9	20.8	17.6	14.7
		**1.5**	**1.4**	**0.8**	**3.3**	**0.3**	**3.3**	**1.2**	**2.5**	**1.4**
	T2J:T3D	18.6	9.9	19.3	16.3	13.0	16.8	20.0	15.7	21.1
		**1.5**	**1.3**	**0.9**	**3.3**	**0.4**	**2.8**	**1.2**	**2.2**	**2.2**
	T1L:T3D	18.2	8.6	11.1	0.0	12.5	3.1	0.0	20.0	27.3
		**0.3**	**0.6**	**0.1**	**0.0**	**0.3**	**0.1**	**0.0**	**0.5**	**0.8**

S1 not included because of uncertainty in where to place gaps.

^a ^Values determined for each pairwise comparison as: # base changes / total such positions × 100.

^b ^Values determined as # of observed changes/ # of positions at which changes could have occurred × 100.

^c ^Upper value indicates proportion of all amino acid substitutions that are conservative or nonconservative (using CLUSTAL W analysis with BLOSUM weighting); semi-conservative substitutions not included. Lower bold value indicates proportion of indicated types of alterations as a percentage of total number of amino acids within whole protein.

The types of amino acid substitutions within each of the prototype isolates' proteins were also examined. Pairwise analyses showed that most substitutions in most proteins were conservative (Table 3). Nonconservative substitutions were relatively rare in most proteins' pair-wise comparisons. For example, comparison of the T1L and T3D μ2 proteins showed none (0.0%) of the 10 amino acid substitutions were nonconservative, and most T1L:T3D comparisons gave low nonconservative substitution values ranging from 0.1–0.5% of total amino acid residues within the respective proteins. However, some genes, most notably M1, M3, and S3, demonstrated higher nonconservative variation, with values approaching 3.5% of total amino acid residues. Most of these higher nonconservative substitution values were observed when T2J proteins were compared to either T1L or T3D proteins. In addition, in many proteins, the majority of nonconservative substitutions were located within the amino-terminal portions (first ~20%) of the respective proteins (data not shown).

The frequencies with which different redundant codons are used to encode certain mammalian amino acids are non-random (reviewed in [57]). This phenomenon is mirrored by different abundances of the complementary tRNA molecules in mammalian cells. For example, CG pairs are underrepresented in mammalian genomes and common in their "rare" codons (see Table 6). A recent study revealed that many RNA viruses of humans display mild deviations from host codon-usage frequencies and that these deviations are more prominent among viruses with segmented genomes [57]. However, reoviruses were not included in that study. By examining reovirus isolates T1L, T2J, and T3D, for which whole-genome sequences are now available, we found that codons that qualify as rare in mammals are not rare in reovirus (Table 6). Moreover, the few codons that qualify as rare in reovirus (ACC, AGC, CCC, CGG, CUC, and GCC; data not shown) are common in mammals. The basis and significance of these deviations remain unknown, but could have impacts on the rates of translation of reovirus proteins. It is perhaps notable in this regard that the four most highly expressed reovirus proteins (μ1, σ3, μNS, and σNS) have the lowest average frequencies of codons that are rare in mammals (Table 6). Thus, incorporation of rare codons into reovirus coding sequences could be a mechanism of dampening the expression of certain viral proteins.

**Table 6:** Codon-usage frequencies in reovirus for eight codons that are rare in mammals

		Frequencies of selected codons in coding sequences of:^a^																
		
			Mammalian genomes			Reovirus genomes			Individual reovirus genome segments (major protein encoded by each)									
					
Codon	AA^b^	Exp^c^	*Mus*	*Bos*	*Homo*	T1L	T2J	T3D	L1 (λ3)	L2 (λ2)	L3 (λ1)	M1 (μ2)	M2 (μ1)	M3 (μNS)	S1 (σ1)	S2 (σ2)	S3 (σNS)	S4 (σ3)
ACG	Thr	**0.25**	0.11	0.13	0.11	**0.23**	**0.30**	**0.24**	**0.17**	**0.28**	**0.22**	**0.27**	**0.17**	**0.16**	**0.30**	**0.38**	**0.26**	**0.20**
CCG	Pro	**0.25**	0.11	0.12	0.11	**0.17**	**0.20**	**0.17**	0.12	**0.20**	**0.15**	**0.27**	**0.20**	**0.14**	**0.18**	**0.25**	0.07	0.11
CGU	Arg	**0.17**	0.09	0.08	0.08	**0.20**	**0.22**	**0.24**	**0.22**	**0.19**	**0.14**	**0.25**	**0.19**	**0.31**	**0.12**	**0.16**	**0.21**	**0.29**
CUA	Leu	**0.17**	0.08	0.09	0.08	**0.15**	**0.13**	**0.14**	**0.18**	**0.13**	**0.14**	**0.19**	0.09	**0.18**	**0.16**	0.09	0.05	**0.16**
GCG	Ala	**0.25**	0.10	0.11	0.11	**0.24**	**0.26**	**0.26**	**0.29**	**0.22**	**0.30**	**0.31**	**0.15**	**0.16**	**0.25**	**0.30**	0.10	**0.29**
GUA	Val	**0.25**	0.12	0.11	0.12	**0.18**	**0.17**	**0.15**	**0.20**	**0.23**	0.12	**0.15**	**0.23**	**0.14**	**0.23**	**0.17**	**0.14**	**0.23**
UCG	Ser	**0.17**	0.05	0.06	0.06	**0.14**	**0.17**	**0.14**	**0.13**	**0.14**	**0.18**	**0.16**	**0.11**	0.03	**0.13**	**0.18**	**0.20**	**0.16**
UUA	Leu	**0.17**	0.06	0.07	0.07	**0.20**	**0.18**	**0.20**	**0.32**	**0.20**	**0.16**	**0.23**	**0.14**	0.07	**0.18**	**0.32**	**0.13**	**0.16**

mean	-	**0.21**	0.09	0.10	0.09	**0.19**	**0.20**	**0.19**	**0.22**	**0.20**	**0.19**	**0.21**	**0.18**	**0.16**	**0.21**	**0.22**	**0.16**	**0.18**

^a ^As fraction of all codons for the particular amino acid. Bold, value higher than that in any of the indicated mammals; underlined, value more than double that in any of the indicated mammals.

^b ^Amino acid encoded by the codon

^c ^Expected frequency if codons for each amino acid are used randomly (assuming equal A, C, G, and U contents and no di- or trinucleotide bias).

## Methods

### Cells and viruses

Reoviruses T1L, T2J, T3D, and T3C12 were Coombs and/or Nibert laboratory stocks. Other reovirus isolates were provided by Dr. T. S. Dermody (Vanderbilt University). Virus clones were amplified to the second passage in murine L929 cell monolayers in Joklik's modified minimal essential medium (Gibco) supplemented to contain 2.5% fetal calf serum (Intergen), 2.5% neonatal bovine serum (Biocell), 2 mM glutamine, 100 U/ml penicillin, 100 μg/ml streptomycin, and 1 μg/ml amphotericin B, and large amounts of virus were grown in spinner culture, extracted with Freon (DuPont) or Vertrel-XF (DuPont), and purified in CsCl gradients, all as previously described [19,58].

### Sequencing the M1 genome segments

All oligonucleotide primers were obtained from Gibco/BRL. Genomic dsRNA was extracted from gradient-purified virions with phenol/chloroform [59]. Strain identity was confirmed by resolving aliquots of each in 10% SDS-PAGE gels and comparing dsRNA band mobilities [60]. Oligonucleotide primers corresponding to either the 5' end of the plus strand or the 5' end of the minus strand were as previously described [40]. Additional oligonucleotides for sequencing were designed and obtained as needed. cDNA copies of the M1 genes of each virus were constructed by using the 5' oligonucleotide primers and reverse transcriptase (Gibco/BRL). The cDNAs were amplified by the polymerase chain reaction [61] and resolved in 0.7% agarose gels [59]. The bands corresponding to the 2.3-kb gene were then excised, purified, and eluted with Qiagen columns, using the manufacterer's instructions. Sequences of the respective cDNAs were determined in both directions by dideoxynucleotide cycle sequencing [62-64], using fluorescent dideoxynucleotides.

Sequences at the termini of each M1 segment were determined by one or both of two methods. For some isolates, sequences near the ends of the segment were determined by modified procedures for rapid amplification of cDNA ends (RACE) as previously described [32,65]. In addition, the sequences at the ends of all M1 segments were determined in both directions by a modification of the 3'-ligation method described by Lambden et al. [66]. Briefly, viral genes from gradient-purified virions were resolved in a 1% agarose gel, and the M segments were excised and eluted with Qiagen columns as described above. Oligonucleotide 3'L1 (5'-CCCCAACCCACTTTTTCCATTACGCCCCTTTCCCCC-3'; phosphorylated at the 5' end and blocked with a biotin group at the 3' end) was ligated to the 3' ends of the M segments according to the manufacterer's directions (Boehringer Mannheim) at 37°C overnight. The ligated genes were repurified by agarose gel and Qiagen columns to remove unincorporated 3'L1 oligonucleotide and precipitated overnight with ice-cold ethanol. The precipitated genes were dissolved in 4 μl of 90% dimethyl sulfoxide. cDNA copies of the ligated M1 genes were constructed by using oligonucleotide 3'L2 (5'-GGGGGAAAGGGGCGTAATGGAAAAAGTGGGTTGGGG-3') and gene-specific internal oligonucleotide primers designed to generate a product of 0.5 to 1.2 kb in length. These constructs were amplified by PCR, purified in 1.5% agarose gels, excised, and eluted as described above. Sequences of these cDNAs were determined with gene-specific internal oligonucleotides and with oligonucleotide 3'L3 (5'-GGGGGAAAGGGGCGTAAT-3') by dideoxy-fluorescence methods.

### Sequence analyses

DNA sequences were analyzed with DNASTAR, DNA Strider, BLITZ, BLAST, and CLUSTAL-W. Phylogenetic analyses were performed using the PHYLIP programs . DNAPARS (parsimony) (Fig. 3) and DNAML (maximum likelihood) (data not shown) produced essentially identical trees. These programs were run using the Jumble option to test the trees using 50 different, randomly generated orders of adding the different sequences. In addition, DNAPENNY (parsimony by brand-and-bound algorithm) generated a tree with the same branch orders as DNAPARS and DNAML. RETREE and DRAWGRAM were used to visualize the tree and to prepare the image for publication. Final refinement of the image was performed with Illustrator. Synonymous and nonsynonymous substitution frequencies were calculated according to the methods of Nei and Gojobori [67] as applied by Dr. B. Korber at . Codon frequencies in the M1 coding sequences were determined using the COUNTCODON program maintained at . Values for codon frequencies in mammalian genomes were obtained from the Codon Usage Database maintained at 

Protein sequence analyses were performed using the GCG programs in SeqWeb version 2 (Accelrys). Multiple sequence alignments were done with PRETTY. Determinations of molecular weights, isoelectric points, and residue counts were done with PEPTIDESORT. Determinations of percent identities in pairwise comparisons were done with GAP. Plots of sequence identity over running windows of different numbers of amino acids (Fig. 4 and data not shown) were generated with PLOTSIMILARITY, and the image for publication was refined with Illustrator (Adobe Systems). In addition, protein sequences were analysed for conservative and nonconservative substitutions by pairwise CLUSTAL-W analyses, using BLOSUM matrix weighting [68].

### SDS-PAGE

Gradient-purified virus and core samples were dissolved in electrophoresis sample buffer (0.24 M Tris [pH 6.8], 1.5% dithiothreitol, 1% SDS), heated to 95°C for 3–5 min, and resolved in a 5–15% SDS-PAGE gradient gel (16.0 × 12.0 × 0.1 cm) [69] at 5 mA for 18 h. Some sets of resolved proteins were fixed, and stained with Coomassie Brilliant Blue R-250 and/or silver [70].

### Immunoblotting

Gradient-purified viral and core proteins were resolved by SDS-PAGE as described above, and sets of resolved proteins were transferred to nitrocellulose membranes with a Semi-Dry Transblot manifold (Bio-Rad Laboratories) according to the manufacturer's instructions. Transfer of all proteins was confirmed by Ponceau S staining. Nonspecific binding was blocked in TBS-T (10 mM Tris [pH 7.5], 100 mM NaCl, 0.1% Tween 20) supplemented with 5% milk proteins, and the membranes probed with polyvalent anti-μ2 antibody (a kind gift from Dr. E. G. Brown, University of Ottawa). Membranes were washed with TBS-T, reacted with horseradish peroxidase-conjugated goat anti-rabbit IgG (Jackson ImmunoResearch Laboratories), and immune complexes detected with the enhanced chemiluminescence system (Amersham Life Sciences) according to the manufacturer's instructions.

### Infections and IF microscopy

CV-1 cells were maintained in Dulbecco's modified Eagles medium (Invitrogen) containing 10% fetal bovine serum (HyClone Laboratories) and 10 μg/ml Gentamycin solution (Invitrogen). Rabbit polyclonal IgG against μNS [71] was purified with protein A and conjugated to Alexa Fluor 488 or Alexa Fluor 594 using a kit obtained from Molecular Probes and titrated to optimize the signal-to-noise ratio. Cells were seeded the day before infection at a density of 1.5 × 10^4^/cm^2 ^in 6-well plates (9.6 cm^2^/well) containing round glass cover slips (18 mm). Cells on cover slips were inoculated with 5 PFU/cell in phosphate-buffered saline (PBS) (137 mM NaCl, 3 mM KCl, 8 mM Na_2_HPO_4 _[pH 7.5]) containing 2 mM MgCl_2_. Virus was adsorbed for 1 h at room temperature before fresh medium was added. Cells were further incubated for 18–24 h at 37°C before fixation for 10 min at room temperature in 2% paraformaldehyde in PBS or 3 min at -20°C in ice-cold methanol. Fixed cells were washed with PBS three times and permeabilized and blocked in PBS containing 1% bovine serum albumin and 0.1% Triton X-100. Antibody was diluted in the blocking solution and incubated with cells for 25–40 min at room temperature. After three washes in PBS, cover slips were mounted on glass slides with Prolong (Molecular Probes). Samples were examined using a Nikon TE-300 inverted microscope equipped with phase and fluorescence optics, and images were collected digitally as described elsewhere [23]. All images were processed and prepared for presentation using Photoshop (Adobe Systems).

## Authors' Contributions

PY and NDK participated equally in designing primers and determining the T2J M1 sequence; TJB, MMA, and JSLP determined the M1 sequences of the T3C12 clone and other labs' T3D clones, as well as factory morphologies of all clones; and all authors participated in writing the manuscript. MLN and KMC are the principal investigators and KMC determined the M1 sequences of the other field isolates and *ts *mutants.
